# An AI-Based Clinical Decision Support System for Antibiotic Therapy in Sepsis (KINBIOTICS): Use Case Analysis

**DOI:** 10.2196/66699

**Published:** 2025-03-04

**Authors:** Juliane Andrea Düvel, David Lampe, Maren Kirchner, Svenja Elkenkamp, Philipp Cimiano, Christoph Düsing, Hannah Marchi, Sophie Schmiegel, Christiane Fuchs, Simon Claßen, Kirsten-Laura Meier, Rainer Borgstedt, Sebastian Rehberg, Wolfgang Greiner

**Affiliations:** 1 Centre for electronic Public Health Research (CePHR), School of Public Health, Bielefeld University, P.O. Box 10 01 31, Bielefeld, D-33501, Germany, 49 521-106-2648; 2 AG 5 - Health Economics and Health Care Management, School of Public Health, Bielefeld University, Bielefeld, Germany; 3 AG Semantic Computing, Technical Faculty, Bielefeld University, Bielefeld, Germany; 4 Data Science Group, Faculty of Business Administration and Economics, Bielefeld University, Bielefeld, Germany; 5 Department of Anaesthesiology, Surgical Intensive Care, Emergency Medicine, and Pain Therapy, Hospital Bielefeld, University Hospital Bielefeld, Bielefeld, Germany; 6 Department of Anaesthesiology, Intensive Care, Emergency Medicine, Transfusion Medicine and Pain Therapy, Campus Bielefeld-Bethel, University Hospital Bielefeld, Bielefeld, Germany

**Keywords:** CDSS, use case analysis, technology acceptance, sepsis, infection, infectious disease, antimicrobial resistance, clinical decision support system, decision-making, clinical support, machine learning, ML, artificial intelligence, AI, algorithm, model, analytics, predictive models, deep learning, early warning, early detection

## Abstract

**Background:**

Antimicrobial resistances pose significant challenges in health care systems. Clinical decision support systems (CDSSs) represent a potential strategy for promoting a more targeted and guideline-based use of antibiotics. The integration of artificial intelligence (AI) into these systems has the potential to support physicians in selecting the most effective drug therapy for a given patient.

**Objective:**

This study aimed to analyze the feasibility of an AI-based CDSS pilot version for antibiotic therapy in sepsis patients and identify facilitating and inhibiting conditions for its implementation in intensive care medicine.

**Methods:**

The evaluation was conducted in 2 steps, using a qualitative methodology. Initially, expert interviews were conducted, in which intensive care physicians were asked to assess the AI-based recommendations for antibiotic therapy in terms of plausibility, layout, and design. Subsequently, focus group interviews were conducted to examine the technology acceptance of the AI-based CDSS. The interviews were anonymized and evaluated using content analysis.

**Results:**

In terms of the feasibility, barriers included variability in previous antibiotic administration practices, which affected the predictive ability of AI recommendations, and the increased effort required to justify deviations from these recommendations. Physicians’ confidence in accepting or rejecting recommendations depended on their level of professional experience. The ability to re-evaluate CDSS recommendations and an intuitive, user-friendly system design were identified as factors that enhanced acceptance and usability. Overall, barriers included low levels of digitization in clinical practice, limited availability of cross-sectoral data, and negative previous experiences with CDSSs. Conversely, facilitators to CDSS implementation were potential time savings, physicians’ openness to adopting new technologies, and positive previous experiences.

**Conclusions:**

Early integration of users is beneficial for both the identification of relevant context factors and the further development of an effective CDSS. Overall, the potential of AI-based CDSSs is offset by inhibiting contextual conditions that impede its acceptance and implementation. The advancement of AI-based CDSSs and the mitigation of these inhibiting conditions are crucial for the realization of its full potential.

## Introduction

Sepsis infections caused by pathogens with antimicrobial resistance (AMR) represent a significant global challenge in health care [1,2]. In 2017, there were 48.9 million new cases of sepsis and 11 million deaths related to sepsis, accounting for 19.7% of all global deaths [3]. In Germany, sepsis incidence increased by an average of 5.7% per year, from 280 cases in 2010 to 370 cases in 2015 per 100,000 individuals [4]. A recent meta-analysis indicated that the 30-day mortality rate for sepsis in Germany was estimated to be 26.5%, which is consistent with the observed rates in North America and Europe [5]. Furthermore, over 1.27 million deaths per year are attributed to AMR worldwide. In 2019, there were 9650 deaths attributable to AMR (mortality rate of 5 per 100,000) and 45,700 deaths associated with AMR (mortality rate of 22 per 100,000) in Germany [6]. Recent data from Germany show heterogeneous trends in AMR proportions of infected patients underscoring the urgent need for enhanced infection prevention measures to limit AMR spread [7]. Inappropriate antibiotic prescribing represents a significant contributing factor [8]. In particular, the prolonged or improper use of nonspecific, broad-spectrum antibiotics is highly problematic, as these antibiotics provide symptomatic relief but also facilitate the development of resistance in other bacteria [9,10].

Clinical decision support systems (CDSSs) provide a solution for promoting targeted and guideline-based antibiotic prescribing [11-13]. CDSSs adopt a variety of forms and are integrated into routine practice. Despite their diverse nature, they share a common objective: to assist medical professionals in identifying the most appropriate form of therapy for each patient, based on existing data and established guidelines, through the use of programmed algorithms or artificial intelligence (AI) [14,15]. In the context of CDSSs, previous studies have demonstrated a decrease in antibiotic prescription rates, more rapid initiation of appropriate antibiotic therapy for patients, and improved clinical outcomes, such as reduced mortality, increased antibiotic-free days, and fewer medical complications [16-19]. However, CDSSs do not always improve clinical practice [20]. For example, there is still insufficient evidence to conclusively show positive effects on therapy duration, dosage, or adherence to clinical guidelines [11,16,21].

Despite the growing body of research on AI-based CDSSs in intensive care units (ICUs) [22,23], there is a notable gap in the implementation of AI tools in routine care in general [24] and the availability of AI-based CDSSs for sepsis in the German health care system. The KINBIOTICS (translated as “AI-based decision support for antibiotic therapy”) project, funded by the German Federal Ministry of Health, aimed to train AI-based algorithms and improve the prediction of suitable antibiotics for sepsis infections on the basis of a comprehensive data set. In addition to the development of an AI-based CDSS, a cross-sectoral resistance observatory and a new rapid test for sequencing the antibiotic genome were developed [25].

Engaging clinicians in the design and development of CDSSs is often suggested as a strategy to enhance the alignment between the system and the needs of its users [26-28]. Interviews and expert groups are approaches used for preimplementation clinician involvement [29]. Insights into clinicians’ views provide valuable information on the barriers and facilitators that affect their willingness to adopt and use CDSSs in practice [30]. By examining clinicians’ perceptions, the study seeks to inform targeted strategies to improve the CDSS’s design, usability, and relevance, thereby promoting more effective and widespread adoption in clinical practice.

This study aimed to perform a use case analysis of a pilot version of the AI-based CDSS within an ICU setting. The objective was to analyze the clinical decision-making processes, and adopt a more comprehensive perspective on the factors that facilitate or hinder the future implementation of such a system.

## Methods

Reporting is based on the Standards for Reporting Qualitative Research (SRQR) [31]. The SRQR checklist is provided in Multimedia Appendix 1.

### Study Design and Setting

A 2-stage base model (random forest at both stages) was developed for the initial therapy using patient data, laboratory data, and clinical data. Subsequently, the model was optimized using a variety of parameters, including the number of decision trees and tree depth (the model specifications and results will be published separately once the AI-based CDSS model has been finalized). The developed model was trained and tested on data from 1 of the 3 participating clinics and then evaluated for robustness using data from a second clinic. Although the performance of the underlying AI model was insufficient in terms of accuracy, specificity and sensitivity at the time of the evaluation, it was crucial at this time to gain insights into the decision-making mechanisms of the physicians. This was undertaken in order to scrutinize the variables included in the initial model and, where necessary, to supplement or adjust them. Accordingly, the preliminary prototype was not modified during the interview phase. However, subsequent to the interviews, the model underwent alterations and is still under development.

**Figure 1.**
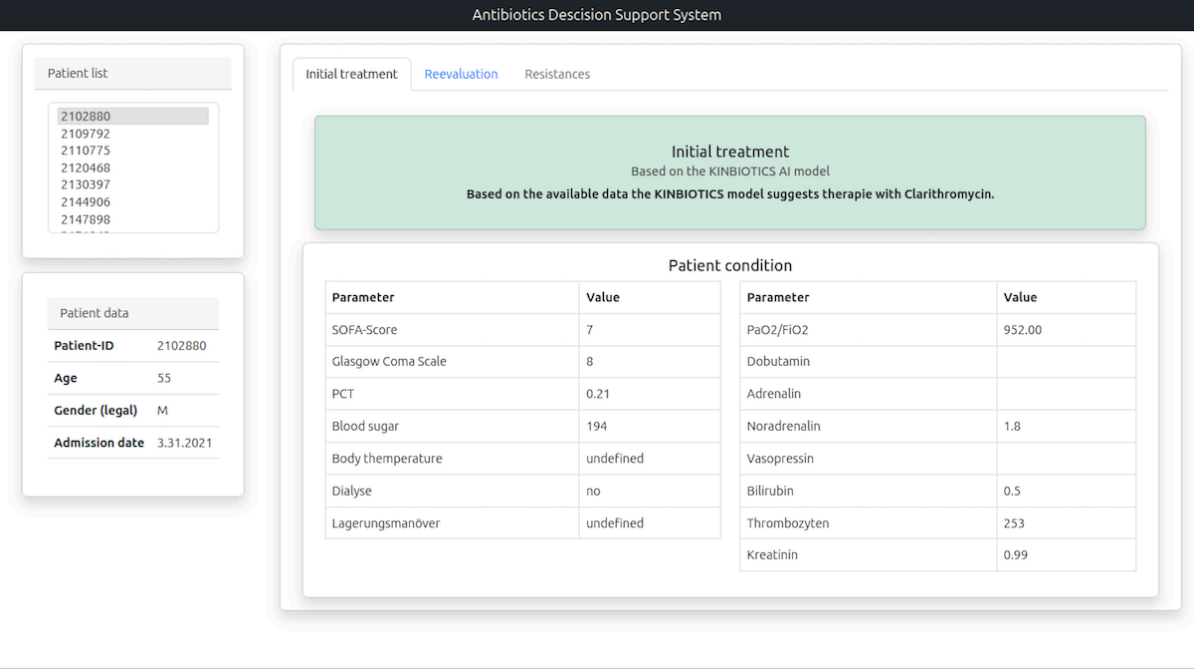
Screenshot of the pilot version of the artificial intelligence–based clinical decision support system.

The pilot version of the AI-based CDSS was evaluated in a 2-stage semistructured qualitative process [32]. With regard to feasibility, physicians were each shown 5 exemplary sepsis patient cases in a desktop version of the CDSS. Cases were selected randomly from the evaluation dataset and were identical for each interviewee. In addition to basic patient information, this contained relevant vital parameters, and the treatment recommendation determined by the AI-based CDSS (Figure 1). This antibiotic therapy recommendation was presented to the physicians both for the admission situation to the ICU (initial treatment; the start of antibiotic therapy) and for a possible therapy correction at the time the microbiological findings were available (re-evaluation; usually 48-72 hours after start of antibiotic therapy). The physicians were informed during the individual interviews that the CDSS is still in a pilot status and that the recommendations are likely not yet reliable. The physicians were asked to assess and evaluate the intensive care situation at both time points as well as the AI-based model recommendation for antibiotic therapy. In addition to the plausibility of the content of the therapy recommendations, the design and layout of the pilot version were evaluated.

Following the individual interviews, physicians from each clinic were interviewed again as part of a focus group interview. The theoretical focus here was on technology acceptance of AI-based CDSSs and the identification of challenges and conductive conditions with regard to future implementation.

### Expert Selection

Expert interviews were conducted with intensive care physicians from the 3 clinical centers involved in the project at the University Medical Center East Westphalia-Lippe. The interviews were conducted on a voluntary basis and the potential participants were selected by the respective project partners in the 3 clinical centers.

### Data Collection

Between October and November 2023, the face-to-face expert interviews and focus groups were conducted. The interview guideline (Textbox 1) for the focus group interviews was developed on the basis of the extended Unified Theory of Acceptance and Use of Technology (UTAUT 2) [33,34]. The UTAUT has recently being used to explain technology adoption among health care practitioners’ intention to use AI-based CDSSs [35].

Textbox 1. **Interview guideline for the focus groups (based on the Unified Theory of Acceptance and Use of Technology 2).**

Opening question

- What were the objectives of today’s meeting?

Questions about the tool

- Please describe your impressions of the KINBIOTICS user interface.
- What information may have been lacking in your medical decision-making process that led to the prescription of antibiotics?
- Please indicate which aspects you consider to be beneficial for a potential application.
- What additional functions would be beneficial to include in the KINBIOTICS user interface to ensure effective utilization in routine clinical practice?

Questions regarding the use of artificial intelligence (AI) in general

- To what extent do digital tools contribute to the processes undertaken in your day-to-day work?

- Please indicate which digital applications you currently use in your professional activities.
- Please indicate which digital health applications you use in your private life.

- Please indicate your opinion on the utilization of AI, and provide your expectations regarding the implementation of AI in healthcare, both in general and on your ward.
- Please describe your previous experience of contact with AI and clinical decision support systems.
- What is the prevailing attitude in your clinical setting, on the ward and among your professional colleagues with regard to the utilization of artificial intelligence in everyday medical procedures?
- In the hypothetical scenario of utilizing such a tool like KINBIOTICS in tomorrow’s clinical decision-making process, what potential challenges might be identified?

Questions about future utilization of a clinical decision support system

- Please describe the benefits you perceive in the utilization of AI and clinical decision support systems in your everyday professional practice.
- In order to apply such a system in medical decision-making, what prerequisites must be met?
- Please indicate whether you identify any additional aspects that should be addressed for use in everyday clinical practice.

The interviews were recorded by audio and subsequently transcribed. After complete transcription, the audio recordings were irrevocably deleted in accordance with the data protection policy.

### Data Analysis

The data analysis of the transcribed interview material was conducted deductively and inductively based on categories according to Kuckartz (2018) [36]. The procedure for the individual interviews primarily followed evaluative content analysis, while the focus groups were analyzed in terms of content structure. To gain as much knowledge as possible, the transcripts of the individual interviews were also analyzed with regard to possible content-structuring findings. The transcripts were analyzed anonymously using the qualitative analysis software MAXQDA (version 2022, VERBI) [37]. The interview analyses and category assignment were carried out independently by 2 researchers (JAD and MK). In the case of discrepancies, a third researcher (DL) was consulted. Categories were adapted or reformulated by the interdisciplinary research team (JAD, MK, DL, SE, and WG).

### Ethical Considerations

Informed consent was obtained before the interviews. A positive ethics vote (November 22, 2021) from the ethics committee of the Medical Association of Westphalia-Lippe and the Westphalian Wilhelms University of Münster was received for the entire project (No. 2021-699-f-S). The interview transcripts were anonymized. The interviewees did not receive any compensation; participation was on a voluntary basis.

## Results

### Sample

A total of 19 individual interviews and 3 focus group interviews were conducted. The distribution of respondents with regard to gender, age, and professional experience was relatively balanced, with 10 (53%) of the respondents identifying as male. In each case, 7 (37%) respondents were aged 30-40 years, and 40-50 years. In total, 5 (26%) respondents had less than 5 years or 11‐20 years of professional experience, 4 (21%) had between 5 and 10 years, and 3 (16%) respondents indicated that they had more than 20 years of professional medical experience. The duration of the expert interviews ranged from 17 to 43 minutes, while the average duration of the focus group interviews was 1 hour.

### Feasibility of the AI-Based Clinical Decision Support System

The approval or rejection of the AI-based antibiotic recommendations was heterogeneous and primarily dependent on 2 factors: first, the plausibility of the AI-based individual therapy (change) recommendations (refer to quotes 1 and 2). It should be noted that not every detailed recommendation was already plausible or medically advisable due to the pilot status of the CDSS respective example cases.

***Quote 1:*** *The system now suggests piperacillin/tazobactam. Of course, this is a very far-fetched suggestion for primary antibiotics when it comes to designing a calculated antibiotic therapy. For me, this would not be the first drug of choice for patients who arrive at the hospital as primary patients, who have been treatment-naïve so far and have not yet received antibiotic therapy [...].*

[Expert 5, Clinical Center 3]

***Quote 2:*** *So, [piperacillin/tazobactam]. Well, difficult [...] with a completely normal PCT [Procalcitonin], I would find it difficult [...] to start directly with such a broad antibiotic*.

[Expert 8, Clinical Center 2]

***Quote 3:*** *[...] So, [...] such a recommendation, I’m very sure, will be malpractice in the next three to five years [...]. This reflects the reality, but this is the worst thing that can happen, to treat a Staphylococcus aureus bacteremia with [piperacillin/tazobactam], that is an absolute no-go.*

[Expert 4, Clinical Center 1]

Second, the evaluation of the recommendations is dependent upon the level of professional experience (quote 3). Accordingly, the more advanced the professional experience, the more confident a recommendation was to be approved or rejected. However, the data indicate that the majority of respondents expressed consensus regarding the CDSS recommendation in each case study (Table 1).

**Table 1.** Assessments of the experts surveyed on the feasibility of the pilot version of the artificial intelligence–based clinical decision support system (N=19).

	CDSS recommendation for initial treatment		CDSS recommendation for re-evaluation	
	Agreeing with the recommendation, n (%)	Rejecting the recommendation, n (%)	Agreeing with the recommendation, n (%)	Rejecting the recommendation, n (%)
Use case 1	14 (78)	4 (22)	12 (67)	6 (33)
Use case 2	11 (61)	7 (39)	1 (5)	18 (95)
Use case 3	3 (16)	16 (84)	2 (11)	16 (89)
Use case 4	15 (83)	3 (17)	14 (78)	4 (22)
Use case 5	13 (76)	4 (24)	12 (71)	5 (29)

A suggestion was made that the pilot CDSS should be expanded to include the integration of combination therapies. The vital parameters and patient information should be included as variables in the statistical model for predicting therapy to include the suspected focus of infection. In addition, it was noted that the data provided for the development of the model and the case studies in the pilot version were incomplete in many instances (Table 1). The physicians encountered significant challenges in evaluating the CDSS recommendation when the fields for data that could be viewed in the application were left empty (quote 4).

***Quote 4:*** *Here again the PCT [Procalcitonin] is missing, that would make it easier for us. We only have [...] indirect information that we have an infection here [...] measured by the thrombocytopenia, and then [...] I can get along with actually using piperacillin and tazobactam primarily here when it comes to the calculated antibiotic therapy [...].*

[Expert 5, Clinical Center 3]

***Quote 5:*** *The [application] is more explanatory than my phone. So, everything is fine in that respect.*

[Expert 3, Clinical Center 3]

From the physicians’ perspective, the primary factor influencing the acceptance of the AI-based CDSS was the time required for completion of the process. If the system is able to produce an evident medication recommendation in a shorter time than the physicians can achieve, a high willingness to use it was reported. There was unanimous feedback that the design and layout of the CDSS are pleasant and easy to understand (quote 5). Furthermore, the majority of respondents indicated a preference for a mobile version of the system for use in everyday clinical practice. This preference was again justified by the availability of digital resources.

### Facilitating and Inhibiting Factors for the Implementation of an AI-Based Clinical Decision Support Systems

The focus group interviews showed that while there is already strong foundational support for digital tools and applications in diagnostics and therapy as part of routine clinical practice, the use of more advanced applications, especially those based on AI, was limited to isolated cases and primarily on a project basis. Previous experiences with CDSSs had a significant impact on expectations regarding the quality of the CDSS, the effort required for integration into everyday medical practice, and the intention to potentially use such a system (quotes 6 and 7).

***Quote 6:*** *I’m thinking about the echocardiography, [...] where the AI has become so good that we let it take over. We do the examination ourselves. And yet the module helps us with that. That’s how I imagine it [here] too.*

[Expert 2, focus group 2]

***Quote 7:*** *Yes, for the colonoscopy [...] we once had [a CDSS]. But the human was faster than the AI. So in that respect [...] rather not.*

[Expert 3, focus group 3]

Nevertheless, the majority of respondents indicated a high level of willingness to adopt AI-based CDSSs (quotes 8 and 9). The option of re-evaluation included in the application was considered to be particularly beneficial.

***Quote 8:*** *I actually found the idea compelling. Because in the early days of my clinical work, even more so than now, the question always arises: am I doing it right now?*

[Expert 1, focus group 2]

***Quote 9:*** *[T]he field is so complex [...] that’s why I think a support tool that works well is very helpful.*

[Expert 3, focus group 1]

As previously noted, the acceptance and implementation of digital solutions is largely dependent on the amount of time available. In addition, the majority of respondents indicated that they are consistently seeking tools that facilitate efficient support in routine clinical practice. Consequently, the respondents exhibited an intrinsic motivation to use CDSSs, which was observed across all age groups and genders, but was particularly pronounced in the “30‐40 years” and “40‐50 years” age groups.

In contrast with the physician’s technology openness as a beneficial factor, the level of digitization in the clinics was found to be heterogeneous. Furthermore, the physicians’ assessment indicated that there was significant potential for improvement in all 3 hospitals (quote 10). The statements indicated that the level of digital infrastructure, including stable internet coverage, exhibited considerable variation not only between the clinics but also between individual wards within the clinics. Overall, there was a consensus that better equipment in terms of digital devices and applications was needed (quote 11). The lack of comprehensive digital and cross-sectoral availability of relevant patient health information was also identified as a barrier to the implementation of AI-based CDSSs and efficient medical treatment in general. This demonstrated that the degree of digitization in everyday clinical practice affects the perceived feasibility of implementing the CDSS and is the most significant barrier to the beneficial factors.

***Quote 10:*** *So, it’s really funny that you can still secure good pens. Because we do so much paper based.*

[Expert 2, focus group 2]

***Quote 11:*** *The problem is that there are far too many individual components that all [...] communicate with each other via interfaces. And in the process, values are lost or the data transfer doesn’t work. And then you end up standing there and yes, the computer hangs.*

[Expert 4, focus group 2]

Furthermore, the existing data basis for the AI-based CDSS model was identified as a significant challenge. The participants were aware that, in addition to the aforementioned lack of digitized patient information, the heterogeneous quality of previous antibiotic administration practice also had an influence on the predictive ability of the recommendation from the CDSS. Furthermore, an increased documentation and justification effort in the event of potential deviation from the CDSS recommendation was also seen as a barrier. However, all participants were aware that such a system would be implemented as a supportive and not a replacement measure, and this was not criticized.

Two relevant recommendations for improvement were derived from the interviews. First, the majority of respondents indicated that applicable guidelines on antibiotic therapy should be incorporated into the specification of the underlying statistical models. Second, there was a recurring suggestion that, alongside the further development of the presented system, the capability to diagnose sepsis infections should also be integrated into a CDSS. Once again, the time to correct diagnosis and subsequent appropriate treatment was identified as the main driver. Suggestions for adapting the tested CDSS comprised the inclusion of quantities and suggestions for the duration of antibiotic administration, in addition to the therapeutic agents themselves (quote 12).

***Quote 12:*** *So, in an ideal world, [...] [AI] can be a huge step forward as a decision-making tool. But [A] the right information has to flow in. And [B] the decisive recommendation [...] is already anticipated here [...]. So, the first step: can this be sepsis? That is an important step. [...] In the second step, a recommendation for initial therapy is helpful [...], yes.*

[Expert 4, focus group 1]

## Discussion

### Principal Findings

Overall, this use case analysis shows various barriers and facilitators for the implementation of AI-based CDSSs for antibiotic therapy in sepsis (Textbox 2). The findings of the expert and focus group interviews indicate that the potential of digitization and, in particular, AI-based tools is expected and viewed in a favorable light by the majority of the physicians. This finding is in line with other research which indicates that clinicians have a predominantly positive perception of AI systems [38-40]. Similarly, the willingness to use these tools is high [41]. In this context, the plausibility of the prediction and the potential time savings are of particular importance. It is evident that the existing contextual conditions represent the most significant obstacle to future implementation. In addition to the basic technical equipment in the clinics, other factors, such as a stable, comprehensive internet supply and the necessary digital availability of data, were also identified as potential barriers to future implementation. Accordingly, the question arises as to what extent clinics are equipped with the digital infrastructure to fully leverage the benefits of AI-based CDSSs. At this point it has to be noted that the digital maturity of German hospitals appears to be comparatively limited [42]. This can be attributed, at least in part, to a lack of financial resources [24,43]. It remains to be seen to what extent hospitals use the transformation fund provided by the German Hospital Future Act and how this can enhance the conditions for the implementation of AI systems.

Textbox 2. **Summary of barriers, facilitators, and recommendations for artificial intelligence–based clinical decision support systems’ feasibility and implementation.**

**Barriers**

- Overall

- Low degree of digitization in everyday clinical practice.
- Lack of comprehensive digital and cross-sectoral data availability.
- Negative previous experiences with clinical decision support systems.

- Use Cases

- Heterogeneous quality of previous antibiotic administration practice limits predictive ability of the artificial intelligence–based recommendations.
- Increased effort to document and justify potential deviations from the clinical decision support system’s recommendation.
- The confidence to approve or reject artificial intelligence–based antibiotic recommendations depends on the level of professional experience.

**Facilitators**

- Overall

- Potential time savings.
- Physician’s technology openness or intrinsic motivation.
- Positive previous experiences with clinical decision support systems.

- Use Cases

- Re-evaluation of clinical decision support system recommendations is considered to be particularly beneficial.
- Pleasant design and layout of the clinical decision support system (easy to understand).

**Recommendations**

- Incorporation of guidelines on antibiotic therapy into the specification of the statistical models.
- Clinical decision support systems should include the integration of combination therapies.
- Statistical model for predicting antibiotic recommendations should include vital parameters and further patient information to identify the suspected focus of infection.
- Besides sepsis treatment, the capability to diagnose sepsis infections should also be integrated into a clinical decision support system.
- Preference for a mobile version of the clinical decision support system.

The medication recommendations by the CDSS were not yet perceived as reliable due to the limited and biased data available for training the AI system. Often, a very broad standard therapy (piperacillin/tazobactam) was recommended. This was mainly due to the fact that this medication occurred considerably more frequently within the learning data set than all other antibiotics. According to the experts, this approach is not absolutely mandatory and sometimes maybe counterproductive in terms of calculated antibiotic therapy. Younger physicians in particular tended to agree with the recommendations. This is a sign that a CDSS for the most common “standard medication” might not be beneficial if not detrimental. This can be confirmed by the physicians’ statements that they would like decision support primarily for medication combinations and for rare antibiotic agents or rare sources of infection. In view of the goal of avoiding the development of resistance by reducing the use of broad-spectrum antibiotics, the current pilot version of the AI system would possibly even promote this risk of further resistance. To avoid this, current guidelines should be incorporated into the model design in addition to retrospective primary data.

The respondents’ call for the upstream integration of sepsis diagnostics in addition to the further development of the existing CDSS appears to be purposeful. A recent study has confirmed initial successes in this regard [44]. Although no clear correlations between sociodemographic characteristics and the willingness to use CDSSs were found in the present evaluation, differences in the approval or rejection of the AI-based CDSS recommendations depending on professional experience were identified. This finding is supported by Lambert et al [45]. It is recommended that future studies investigate the degree to which professional experience and other sociodemographic variables exert an influence on technology acceptance and the willingness to use CDSSs. In this regard, using the UTAUT would be appreciated [35].

Generally, the following challenges associated with the use of AI in health care should not be overlooked. For example, automation bias, in which health care providers may develop an overreliance on AI recommendations, potentially reducing their clinical vigilance and leading to oversights in patient care, needs to be considered [46]. Furthermore, AI-driven CDSSs may unintentionally propagate biases present in training datasets, resulting in discriminatory recommendations that may disadvantage certain demographic groups [47]. Another key concern is the ambiguity and complexity of liability; it remains unclear who is legally responsible if an AI-influenced decision contributes to a negative patient outcome [48]. Furthermore, previous research has highlighted the potential negative impact of a lack of user involvement in the development of CDSSs on the willingness to use them, even when the system quality is optimal [26]. One of the key strengths of the study is the early involvement of future users. The participatory approach highlights the need for involvement. Consequently, given the significant lack of engagement with CDSS interventions reported within the literature [49], future studies should consider the involvement of users from the earliest stages of development [26,28,50]. The findings of the present study confirm that in addition to personal factors, the plausibility of the recommendations is the most decisive factor determining the willingness to use the tool [51]. Further development of the pilot version or similar systems is therefore crucial for implementing and exploiting the potential benefits of AI. Furthermore, the feedback from the physicians participating in this study provided valuable insights into additional variables that were subsequently integrated into the further development of the CDSS, following the completion of the interviews.

### Limitations

Several limitations must be acknowledged in the evaluation of the use case presented in this study. First, the efficacy of the CDSS was clearly limited due to the rather small amount of data used for training. The findings suggest that the CDSS may offer less benefit for physicians during the initial therapy phase compared with its use in the context of therapy re-evaluation. At this stage, it was not feasible to accurately predict the most appropriate antibiotic, one that would minimize treatment changes and side effects, based on the currently available data. The acceptance of AI-based interventions heavily depends on the plausibility and relevance of therapy recommendations, which ultimately impacts their efficacy. Therefore, further development and refinement of AI-based CDSSs are essential, along with an expansion of the digital data infrastructure.

In addition, it is important to consider that the evaluation was based on a qualitative interview study, which comes with inherent limitations. The voluntary nature of participation may introduce self-selection bias, potentially leading to an overestimation of the participants’ openness to technology and their acceptance of AI-based CDSSs. This limitation suggests that the findings may not be fully generalizable to the broader population of health care professionals.

### Conclusion

This pilot study of an AI-based CDSS indicates that further development is needed to achieve the original goals of minimizing switches in antibiotic prescriptions and reducing the reliance on broad-spectrum antibiotics. Relying solely on retrospective data from past care practices does not seem to be an effective strategy for meeting these objectives. In addition, it is essential to address the key challenges identified in this study to enable the successful integration of AI into clinical settings. While the respondents generally showed a positive attitude toward the use of AI-based CDSSs, a more comprehensive evaluation of technology acceptance among health care professionals can only be conducted when a version with realistic, guideline-compliant recommendations is available. Early engagement of users in the development process proves beneficial, not only for refining the CDSS but also for fostering acceptance among medical practitioners.

## Supplementary material

10.2196/66699 Multimedia Appendix 1 Standards for Reporting Qualitative Research checklist.

## Abbreviations

AI: artificial intelligence
AMR: antimicrobial resistance
CDSS: clinical decision support system
ICU: intensive care unit
SRQR: Standards for Reporting Qualitative Research
UTAUT: Unified Theory of Acceptance and Use of Technology
